# Exploring the Pathological Mechanism of Bladder Cancer Based on Tumor Mutational Burden Analysis

**DOI:** 10.1155/2019/1093815

**Published:** 2019-08-25

**Authors:** Yao Ma, Xiao-Fei Feng, Wan-Xia Yang, Chong-Ge You

**Affiliations:** ^1^Laboratory Medicine Center, Lanzhou University Second Hospital, Lanzhou 730030, China; ^2^Department of Orthopaedics, Lanzhou University Second Hospital, Lanzhou 730030, China; ^3^Key Laboratory of Osteoarthritis of Gansu Province, Lanzhou University Second Hospital, No. 82 Cuiyingmen, Lanzhou, Gansu 730030, China

## Abstract

Although immunotherapy has progressed in the treatment of bladder cancer, some patients still have poor prognosis. New therapeutic targets are eager to be discovered to improve the outcomes of bladder cancer. With the development of high-throughput sequencing and tumor profiling, potential tumor biomarkers were identified. Through the interpretation of related data from the Cancer Genome Atlas database (TCGA), some key genes have been discovered to drive the development and prognosis of urinary bladder neoplasm. On account of the success of immunotherapy in many cancer types, we established the relationship between tumor mutation burden and immune microenvironment of bladder cancer and found the changes of several immune cells in this disease. Based on the understanding of the bladder tumor genome and immune environment, this study is supposed to provide new therapies for the treatment of bladder neoplasm.

## 1. Introduction

Bladder cancer is a major malignant tumor with a high incidence in the urinary system. It is one of the most common cancers worldwide [1], which seriously affects people's quality of life and increases the economic burden of society. According to the 2018's report, there are 549393 new cases of urinary bladder neoplasm and 199,922 deaths globally [2]. Compared to previous data, the morbidity and mortality of this disease are persistently increasing. Urothelial carcinoma is the most common histopathological type of bladder tumor [3]. According to the clinical staging criteria, nonmuscle invasive bladder cancer, muscle invasive bladder cancer, and metastatic urothelial carcinoma have different treatment methods and therapeutic effects. Primary noninvasive bladder cancer can be remedied by transurethral resection or intravesical injection [4, 5]. Nevertheless, cisplatin-based chemotherapy was first used for muscle-infiltrating urothelial carcinoma before radical cystectomy or the option of bladder preservation [6, 7]. Combination chemotherapy with cisplatin is the common treatment choice for patients with metastatic urothelial carcinoma [8], and most patients are limited by first-line cisplatin chemotherapy because of impaired renal function and other complications [9]. It is exciting to note that immunological checkpoint inhibitors are increasingly being utilized to treat refractory or non-platinum-compliant bladder cancer and have improved the treatment prospects for this disease [10].

Tumor mutational burden (TMB) is defined as the total number of somatic missense mutations present in the baseline tumor sample and it has been a biomarker of tumorigenesis and immune response [11, 12]. The gradual accumulation of somatic mutations leads to the formation of neoantigens that activate T cell immunogenicity to inhibit tumor cells [13]. Tumors such as non-small-cell lung cancer, melanoma, and bladder tumor with high TMB often produce new antigens leading to enrichment of immune cells. Therefore, tumor immunological checkpoints have beneficial therapeutic effects [14–18]. Morales et al. found the activity of Bacillus Calmette-Guerin in the treatment of nonmuscle invasive bladder cancer, which has been called immunoreactive tumors [19, 20]. Inspired of this work, a novel strategy was composed in this work for clinical treatment by exploring the relationship between TMB and immune cells in bladder neoplasm. First, data related to malignant tumor of urinary bladder in TCGA database were collected and the TMB values were calculated then. At last, the relationship between TMB and patient survival in bladder cancer was constructed. After then, the microenvironment of bladder tumor was studied and new tumor immunotherapy for the diagnosis and treatment of this disease was explored.

## 2. Subjects and Methods

### 2.1. Genomic Data of Bladder Cancer

Somatic mutation, gene expression, and clinical data for 412 GC samples were downloaded from TCGA, which is a public database integrating multiple cancer resources [21]. The Simple Nucleotide Variation data determined by the TCGA is called the somatic variation of the MAF file. For RNA-seq expression data, the level 3 RNA-seq FPKM dataset was downloaded.

### 2.2. Extraction of Mutational Signature and Clinical Data

The maftools package in R language is used for the visualization and analysis of mutated spectral data [22]. To calculate the TMB per megabase, the total number of mutations counted is divided by the size of the coding region of the targeted territory. Here, 38 Mb was utilized as the exome size [23]. The survival time, survival status, gender, tumor stage, and TNM stage data of patients were extracted from the clinical data. The survival curve was then plotted using R language, as well as the correlation between TMB and clinical data.

### 2.3. Differential Expression Genes Analysis

After the gene expression data is obtained, pretreatment is performed. Then, in order to calculate the TMB value, all the samples were split into high-TMB and low-TMB groups according to the median value of the TMB. The limma package was used to find the differentially expressed genes (DEGs) between the two groups [24]. Take an average when a gene is present multiple times, and take |log⁡2 FC| > 1 and FDR < 0.05 as the cutoff criterion. Heat maps of DEGs were constructed using the heatmap package in R program.

### 2.4. Gene Ontology and Pathway Enrichment Analysis

Gene Ontology (GO) provides three interpretations of functional genomics, as molecular functions, biological processes, and cellular components [25]. The function of Kyoto Encyclopedia of Genes and Genomes (KEGG) is to form a network of molecules that interact with a certain group of genes in the genome [26]. They both play an indispensable role in the process of bioinformatics analysis. The DEGs for GO and KEGG pathway enrichment were analyzed using the clusterProfiler package [27], with p-value < 0.05 and q-value < 0.05 as statistically significant.

### 2.5. Prognosis

Gene Expression Profiling Interactive Analysis (GEPIA, http://gepia.cancer-pku.cn/) is an open website consisting of seven sections: general, differential genes, expression DIY, survival, similar genes, correlation, and PCA [28]. In the current study, GEPIA was used to explore the association of DEGs with overall survival in patients with bladder cancer. Patients were divided into the high and low expression groups according to the median value of gene expression, with p-value < 0.05 as statistically significant.

### 2.6. Relationship between TMB and Immune Cells

To gain insight into the composition of urinary bladder neoplasm immune cells, the mRNA expression matrix was corrected and CIBERSORT was used to estimate the proportion of 22 human immune cell subsets [29]. The LM22 dataset which was downloaded from the CIBERSORT website (https://cibersort.stanford.edu/download.php) comprises 22 different immune cell types. 1000 permutations and p-value < 0.05 were set as the criteria for the successful deconvolution of a sample [30].

## 3. Result

### 3.1. Gene Mutation Type and Sample Mutation Information

**Figure 1:**
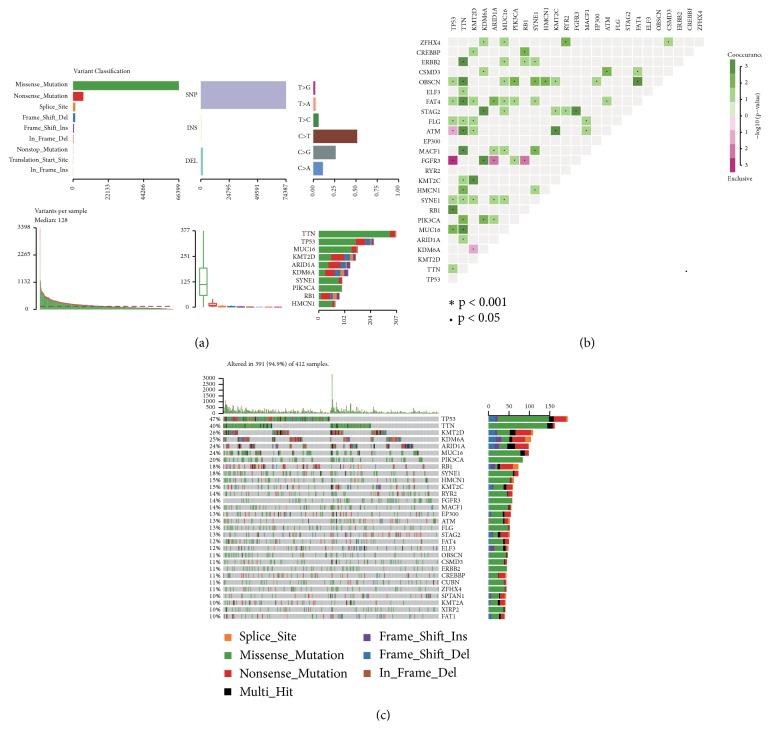
Visualization of somatic disorders from bladder cancer. (a) Cohort summary plot displaying distribution of variants according to variant classification, type, and SNV class. Bottom part (from left to right) indicates mutation load for each sample, variant classification type. A stacked barplot shows top ten mutated genes. (b) Mutually exclusive and cooccurring gene pairs in bladder cancer displayed as a triangular matrix. Green indicates tendency toward cooccurrence, whereas pink indicates tendency toward exclusiveness. (c) Waterfall plot displaying the somatic landscape of BLCA cohort. Genes are ordered by their mutation frequency, and the type of mutation is shown in the comment bar (bottom).

The bladder neoplasm mutation data were analyzed by the Maftools package. The most common mutations in the malignant tumor of urinary bladder are missense mutations. The mutation type accounts for the majority of SNPs. The C > T transition in the SNV classification accounts for the largest part, followed by the C > G transition. At the same time, the top 10 genes with the most mutations in urinary bladder neoplasm were obtained (see Figure 1(a)). Recent studies have pointed out that dysregulated genes in cancer are mutated in a mutually exclusive or cooccurring manner [31, 32], and results of mutually exclusive or simultaneous gene mutations in the disease were obtained (see Figure 1(b)). For example, mutant TP53 and mutant FGFR3 are mutually exclusive (p-value < 0.001). Mutant TP53 and mutant RB1 coexist (p-value < 0.001). Mutant TTN coexists significantly with mutant ERBB2, OBSCN, FAT4, ATM, MACF1, and MUC16 (p-value < 0.001). A waterfall map is drawn by a significant mutant gene which is identified by the MutSigCV algorithm [33] (see Figure 1(c)). This map shows the details of this mutation in 319 samples, which are mainly missense mutations.

### 3.2. TMB and Clinical Relevance

**Figure 2:**
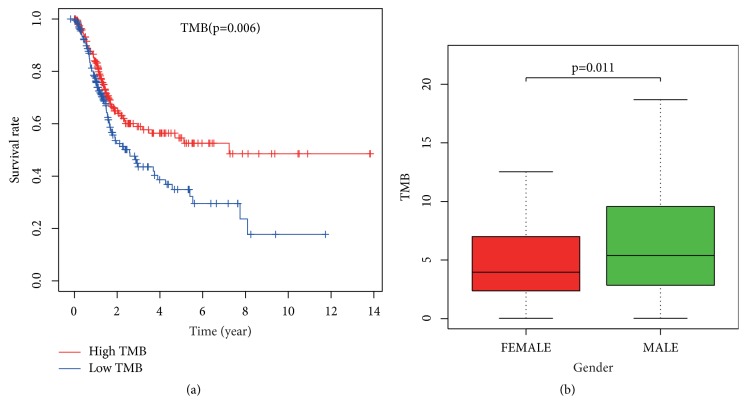
(a) Correlation between tumor mutation burden and survival rate. (b) The relationship between tumor mutation burden and gender.

After calculating the TMB value, there were 208 samples with low-TMB based on the median value and 203 samples with high-TMB. Survival curves were drawn using survival time and survival status of patients (see Figure 2(a)). It was concluded that the survival rate of the group with high-TMB was significantly higher than that of the group with low-TMB (p-value < 0.006). Subsequently, information on patients' gender, pathological stage, and TNM stage was extracted to investigate that whether they are correlated with TMB. The results showed that TMB was associated with gender (p-value < 0.011) (see Figure 2(b)). But there was no statistical significance among pathological stage and TNM stage with TMB.

### 3.3. Identifying Genes Associated with TMB

**Figure 3:**
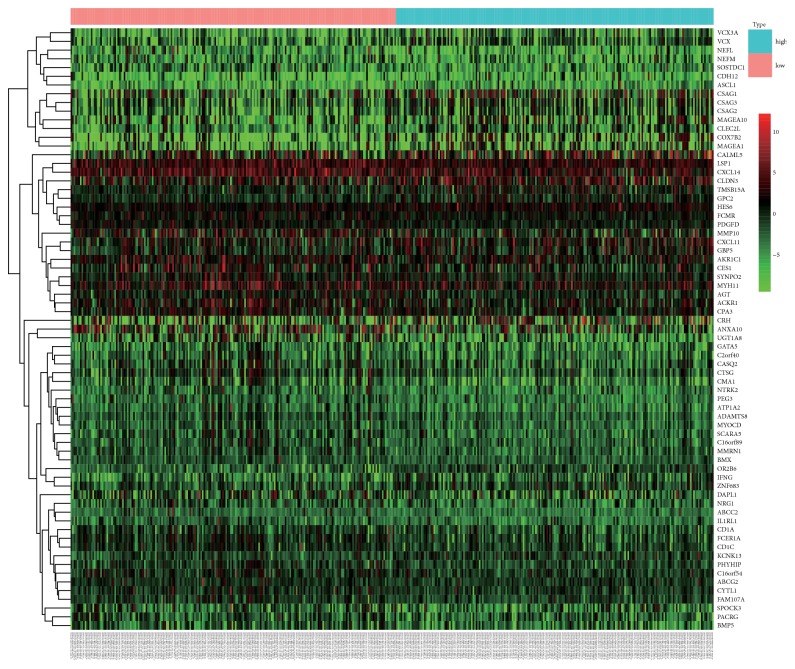
Heat map of 69 differentially expressed genes. Blue: high-TMB group. Pink: low-TMB group. Red: upregulated gene. Green: downregulated genes.

A high-TMB and a low-TMB group were obtained and 69 DEGs were identified. Among them, 46 genes were highly expressed in the low-TMB group, while 23 genes were highly expressed in the high-TMB group. These DEGs were mapped into heat maps (see Figure 3).

### 3.4. Analysis of Differentially Expressed Genes in GO and KEGG

To further explore the biological functions and mechanisms of differentially expressed genes, analysis packages in R software for GO and KEGG were utilized. The GO results were enriched into 19 terms, including primarily hormone metabolic process, regulation of systemic arterial blood pressure by renin-angiotensin, angiotensin maturation, regulation of angiotensin levels in blood, and endocrine process (see Table 1). Besides, KEGG pathway enrichment indicated that four differentially expressed genes were involved in the “Renin-angiotensin system” pathway.

### 3.5. Survival Analysis

**Figure 4:**
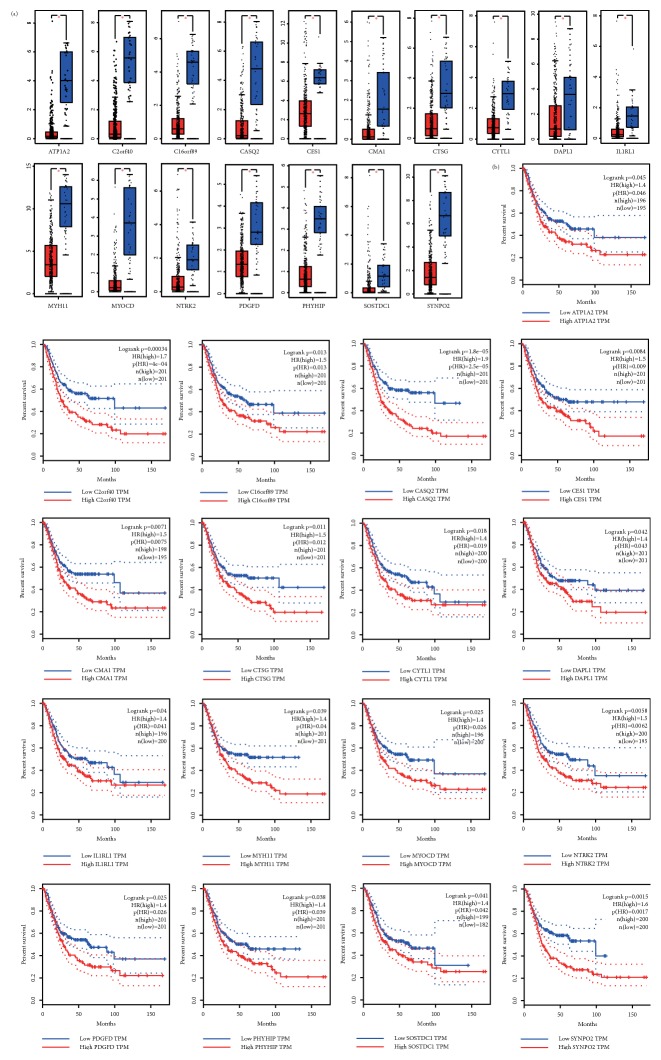
(a) Expression of 17 genes in tumor and normal tissues was significantly different; all had statistical significance. (b) Kaplan-Meier survival analysis of 17 genes in bladder cancer.

To find candidate genes that may affect survival outcomes, a survival analysis of all DEGs was performed. Using p-value < 0.05 as a significant level when exploring DEGs and survival values in the GEPIA online database, 17 genes were found to be significantly associated with overall survival in patients with bladder cancer. Increased mRNA expression of these genes reduced the overall survival of the patient (see Figure 4). They may have played a significant role in the pathogenesis of the disease. Thus these genes could be potential biomarkers for bladder neoplasm prognosis. The expression of these genes in tumors and normal tissues was also analyzed.

### 3.6. Relationship between TMB and Immune Microenvironment

**Figure 5:**
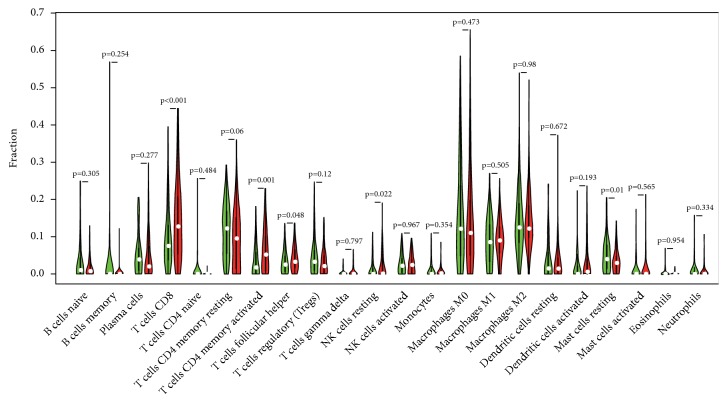
Violin diagram of the relationship between tumor mutational burden and immune cells. Green: low-TMB. Red: high-TMB.

After the previous processes then data on the expression of immune cells in 22 bladder cancer samples were obtained. With p-value < 0.5 as the cut-off standard, 87 samples with low-TMB and 99 samples with high-TMB were obtained. The vioplot package in R language was used to draw a violin map (see Figure 5). Compared with the low-TMB group, samples with high-TMB had higher levels of T cells CD8, T cells CD4 memory activated, and NK cells resting, while Mast cells resting was lower.

## 4. Discussion

Although cisplatin-based combination chemotherapy is still the first-line treatment for urothelial carcinoma, fortunately, the presence of immune checkpoint inhibitors provides new therapeutic targets for patients who are unsuitable and refractory to cisplatin. Since 2016, five new immune checkpoint inhibitors have been approved by the US Food and Drug Administration for the treatment of refractory urothelial carcinoma [34]. Studies have demonstrated that there is a relationship between TMB and immunotherapy responsiveness [11, 35–38]. At the same time, TMB is closely linked to the prognosis of cancer patients [39, 40]. Predicting the efficacy of immunological checkpoint inhibitors through a comprehensive evaluation of tumor microenvironment has become a hot research direction. Our results show that high-TMB often has a relatively favorable living condition, and there is also a correlation between TMB and tumor infiltrating immune cells.

The purpose of our study was to screen and identify biomarkers related to bladder cancer prognosis through a series of bioinformatics analysis of relevant data in the TCGA database. 69 DEGs were screened by dividing the samples into high-TMB and low-TMB. To investigate the molecular mechanisms involved in these DEGs, GO and KEGG pathway analysis was performed and it is found out that DEGs are commonly involved in functional terms and pathways which are related to urinary bladder neoplasm progression. For example, melatonin can inhibit the growth and invasion of bladder tumor cells [41]. Angiotensin type 2 dysregulation affects the proliferation and apoptosis of bladder cancer cells [42]. Kaplan-Meier survival analysis showed that 17 genes were significantly associated with the overall survival of malignant tumor of urinary bladder, and these genes can be treated as novel targets for the treatment of bladder neoplasm. Finally, the results showed that CD4 and CD8 T cells increased in the high-TMB group of bladder cancer. Studies have demonstrated that CD4 and CD8 T cells can inhibit tumor development and form tumor immunogenicity [43]. CD8 T cell is a key player in antitumor immunity [44, 45]. Th1-cytokines derived from CD4 lymphocytes have a role in preventing tumor progression [46]. Resting NK cells are highly expressed in urinary bladder neoplasm high-TMB samples; Turin I et al. indicate that resting NK cells are not toxic to tumor cells, whereas NK cells activated by IL-2 or IL-15 have tumor suppressor the function [47]. IL-2 activated NK cells have antitumor effects [48, 49]. The level of resting mast cells is high in bladder cancer low-TMB samples. Interestingly, mast cells activated by different stimuli release pro- and antitumor substances [50]. Our study found that CD4 and CD8 T cells, as well as NK cells, may be major players in antitumor immunity in bladder cancers with high-TMB.

However, the present study has a few limitations. First of all, the data used in this study was from public databases, not generated by ourselves. Second, there may be other factors caused by genetic mutations which may affect the accuracy of the results. Therefore, further research based on a larger sample size is needed to confirm our results.

## 5. Conclusions

Understanding and identifying the molecular basis for genesis of diseases are central to the development of novel therapies. This study provides a bioinformatics analysis of DGEs, which may be associated with the development and progression of bladder tumors. Our results may contribute to understanding the underlying molecular mechanisms of bladder tumors. 20 genes related to prognosis, which are hoped to be used as targets for urinary bladder neoplasm treatment, were screened in this work. Immune cells related to TMB in bladder neoplasm, which is expected to promote the clinical application of existing immunotherapy programs in this disease, were also analyzed. Then, the results of this work will accelerate the immunotherapy of bladder cancer to help treatment and improvement of the survival and prognosis of patients.

## Figures and Tables

**Table 1:** GO enrichment results.

Term	Function	P-value	Q-value
GO:0042445	Hormone metabolic process	6.42E-07	0.000836
GO:0003081	*Hormone metabolic process*	1.62E-06	0.001055
GO:0002003	Angiotensin maturation	5.81E-06	0.002077
GO:0002002	Regulation of angiotensin levels in blood	7.73E-06	0.002077
GO:0050886	Endocrine process	9.05E-06	0.002077
GO:0001990	Regulation of systemic arterial blood pressure by hormone	9.56E-06	0.002077
GO:0003073	Regulation of systemic arterial blood pressure	1.27E-05	0.002362
GO:0003044	Regulation of systemic arterial blood pressure mediated by a chemical signal	2.17E-05	0.00353
GO:0008217	Regulation of blood pressure	2.78E-05	0.004032
GO:0001991	Regulation of systemic arterial blood pressure by circulatory renin-angiotensin	3.35E-05	0.004361
GO:0008202	Steroid metabolic process	9.10E-05	0.010781
GO:0016486	Peptide hormone processing	0.000166	0.018034
GO:0045445	Myoblast differentiation	0.000189	0.018971
GO:0016101	Diterpenoid metabolic process	0.000257	0.023412
GO:0071466	Cellular response to xenobiotic stimulus	0.000269	0.023412
GO:0006721	Terpenoid metabolic process	0.00041	0.032581
GO:0006939	Smooth muscle contraction	0.000425	0.032581
GO:1903789	Regulation of amino acid transmembrane transport	0.000489	0.035412
GO:0048018	Receptor ligand activity	0.000204	0.038487

## Data Availability

Mutation data, RNA-seq, and clinical data of bladder cancer in our study were obtained from TCGA (https://portal.gdc.cancer.gov/). Data for survival analysis and gene expression in bladder tumors and normal samples were derived from GEPIA (http://gepia.cancer-pku.cn/).
